# Durability of Semaglutide Effects After Treatment Discontinuation in HIV-Associated Lipohypertrophy: A Randomized, Double-Blind, Placebo-Controlled Phase 2b Clinical Trial

**DOI:** 10.1093/ofid/ofag631

**Published:** 2026-10-05

**Authors:** Ruth O Adekunle, Qian Wu, Abdus Sattar, Kianoush Ansari-Gilani, Danielle Labbato, Aaron A Fletcher, Aidan D McComsey, Allison Ross Eckard

**Affiliations:** Medical University of South Carolina, Department of Medicine and Pediatrics, Division of Infectious Disease, Charleston, South Carolina, USA; Case Western Reserve University, Department of Population and Quantitative Health Sciences, Cleveland, Ohio, USA; Case Western Reserve University, Department of Population and Quantitative Health Sciences, Cleveland, Ohio, USA; University Hospitals Cleveland Medical Center, Department of Medicine, Division of Infectious Disease, Cleveland, Ohio, USA; University Hospitals Cleveland Medical Center, Department of Medicine, Division of Infectious Disease, Cleveland, Ohio, USA; University Hospitals Cleveland Medical Center, Department of Medicine, Division of Infectious Disease, Cleveland, Ohio, USA; University Hospitals Cleveland Medical Center, Department of Medicine, Division of Infectious Disease, Cleveland, Ohio, USA; Saint Ignatius High School, Cleveland, Ohio, USA; Medical University of South Carolina, Department of Medicine and Pediatrics, Division of Infectious Disease, Charleston, South Carolina, USA

**Keywords:** GLP-1RA, HIV, semaglutide, treatment discontinuation, weight regain

## Abstract

**Background:**

Human immunodeficiency virus (HIV)–associated lipohypertrophy is characterized by excess central and visceral adiposity and is associated with increased cardiometabolic risk among people with HIV (PWH). In a previously reported randomized trial, semaglutide significantly reduced body weight, regional adiposity, and insulin resistance over 32 weeks of active treatment. However, the durability of these benefits after treatment discontinuation is unknown.

**Methods:**

We conducted a randomized, placebo-controlled trial in PWH with HIV-associated lipohypertrophy. Participants were randomized to semaglutide or placebo for 32 weeks, followed by discontinuation of study drug and observational follow-up through week 56. The current analysis investigated effects of semaglutide at week 56, as compared to baseline and week 32. Outcomes included adipose tissue quantity by body compartment. Analyses were performed using intention-to-treat principles. This trial was registered at ClinicalTrials.gov (NCT04019197) and is complete.

**Results:**

Of 108 participants initially randomized, 92 completed the 32-week intervention and 80 participants (39 semaglutide, 41 placebo) completed the posttreatment phase. Participants previously assigned to semaglutide experienced increases in body weight, body mass index (BMI), waist circumference, total body fat, and visceral adipose tissue between weeks 32 and 56, accompanied by worsening glycemic indices and insulin resistance. Despite this rebound, net reductions in body weight, BMI, and waist circumference were retained at week 56 compared with baseline. Placebo-treated participants demonstrated relative stability across outcomes.

**Conclusions:**

Discontinuation of semaglutide in PWH with lipohypertrophy resulted in partial loss of improvements in weight, adiposity, and metabolic parameters. These findings support that sustained pharmacologic exposure is likely needed to achieve long-term benefit.

## Teaser

Semaglutide discontinuation in people with HIV and lipohypertrophy led to partial reversal of weight, adiposity, and metabolic improvements, though modest reductions persisted. Findings suggest limited durability after withdrawal and support a chronic disease model requiring sustained therapy to maintain benefit.

Human immunodeficiency virus (HIV-1)–associated lipohypertrophy remains a clinically significant complication among people with HIV (PWH), characterized by excess central and visceral adiposity and associated with increased cardiometabolic risk. Glucagon-like peptide-1 receptor agonists (GLP-1RAs) have emerged as promising therapies for addressing excess adiposity and associated metabolic complications. In our previously reported randomized controlled trial, 32 weeks of once-weekly semaglutide significantly reduced abdominal visceral adipose tissue (VAT), total and regional body fat, liver fat, body weight, and insulin resistance in PWH with HIV-associated lipohypertrophy [1]. These findings suggest that GLP-1RAs may provide an important therapeutic option for a condition for which effective pharmacologic interventions remain limited [1].

An important unanswered question is whether these improvements persist after GLP-1RA therapy is discontinued. Increasing evidence from the general population suggests that continued pharmacologic exposure may be necessary to maintain the weight and metabolic benefits of GLP-1RAs [2, 3]. Following semaglutide withdrawal in the STEP 1 trial extension, participants regained approximately two-thirds of their prior weight loss over the subsequent year, accompanied by deterioration in several cardiometabolic risk factors [2]. However, changes in body weight alone may not fully characterize the consequences of treatment withdrawal. The distribution and composition of regained weight, including reaccumulation of visceral and ectopic adipose tissue and changes in lean mass, may be particularly relevant to long-term cardiometabolic risk.

Data regarding the durability of GLP-1RA effects after discontinuation among PWH remain limited. In a recent single-arm study of 49 PWH with metabolic dysfunction–associated steatotic liver disease, participants regained a mean of 2.9 kg during the 24 weeks following semaglutide discontinuation, accompanied by increases in waist circumference and fasting glucose [3]. Although HIV-associated lipohypertrophy has historically been described as a distinct phenotype of central and visceral adiposity, contemporary obesity and antiretroviral therapy (ART)–associated weight gain may overlap substantially with this phenotype, making their individual contributions difficult to distinguish. Thus, rather than presupposing a distinct response to GLP-1RA withdrawal, additional data are needed to determine whether the patterns observed in the general population extend to PWH with central adiposity. Furthermore, changes in regional adipose tissue distribution, ectopic fat, lean mass, and broader cardiometabolic parameters after semaglutide discontinuation have not been comprehensively evaluated in this population. Therefore, the purpose of this study was to evaluate the durability of semaglutide and trajectory of treatment effects of these parameters 24 weeks after treatment discontinuation in PWH with HIV-associated lipohypertrophy.

## METHODS

### Study Design

This was the prespecified 56-week follow-up of a randomized, double-blind, placebo-controlled phase 2b clinical trial conducted at University Hospitals Cleveland Medical Center (Cleveland, Ohio, USA). Full details of the study design, including inclusion criteria, randomization, masking, and procedures, have been previously published [1]. Participants were randomized 1:1 to receive either semaglutide or matching placebo for 32 weeks. At the end of the interventional phase (week 32), semaglutide treatment or matching placebo was stopped and participants entered a 24-week off-treatment observational phase. Participants returned at week 56 for assessments identical to those performed at baseline and week 32. All participants, investigators, and providers remained blinded until study completion (unless unmasking was deemed necessary for individual safety reasons).

Institutional Review Board IORG000040 approved the study; the study was registered on ClinicalTrials.gov (NCT04019197). All participants provided written informed consent. Participants received the study drug free of charge during the study. The protocol used for this trial is available in the Supplementary material.

### Participants

Eligibility criteria have been previously described in detail [1]. In brief, participants were adults with HIV-1 who had received ART for ≥1 year, were on a stable ART regimen for ≥12 weeks, and had maintained HIV-1 RNA <400 copies/mL for ≥6 months. HIV-associated lipohypertrophy was defined by participant-reported increased abdominal girth following ART initiation together with sex-specific waist circumference and waist-to-hip ratio criteria. Participants were also required to have a body mass index (BMI) ≥25 kg/m^2^ and no known diabetes. Full inclusion and exclusion criteria are provided in the primary trial publication. Participants who completed the 32-week interventional phase were eligible for the posttreatment follow-up through week 56.

### Study Procedures and Outcome Measures

As previously described, participants underwent comprehensive clinical and fasting laboratory assessments at each study time point [1]. Clinical evaluations included standardized anthropometric measurements, fasting laboratory evaluations, 2-hour oral glucose tolerance testing (OGTT), dietary intake, and physical activity. Ten-year atherosclerotic cardiovascular disease risk (ASCVD) was estimated using American College of Cardiology's ASCVD Risk Estimator Plus. HIV-1 RNA and CD4^+^ T-cell counts were obtained as part of standard-of-care practice.

Body composition was assessed using whole-body dual-energy X-ray absorptiometry (DXA) and noncontrast helical computed tomography (CT) of chest and abdomen with standardized protocols [3]. Whole-body CT was used to quantify fat mass (total body, limb, trunk) and lean body mass. Single-slice CT was used to estimate psoas muscle area and density, liver fat, and pericardial fat using previously described methods [4, 5].

All biomarkers assessed in the original analysis were included in this current analysis, including glycated hemoglobin (HbA1c), fasting and 2-hour glucose and insulin, homeostatic model assessment of insulin resistance (HOMA-IR), lipid parameters, blood pressure, and estimated 10-year ASCVD risk [2].

### Statistical Analyses

Between-group comparisons were performed using *t*-tests or Wilcoxon rank-sum tests, as appropriate. Generalized estimating equations (GEEs) were used to model the effects of semaglutide on outcomes over time, accounting for repeated measures and covariates. We specified a Gaussian family, an identity link, and unstructured correlation for within-group repeated measurements in modeling an outcome using GEEs. Models included treatment, time, and their interaction. For significant interactions, linear combinations of regression coefficients were used to estimate treatment effects.

Appropriate transformations were applied to outcome variables with skewed distributions prior to forming the GEE models. For highly-skewed variables where transformations could not create a symmetric distribution, simultaneous quantile (median) regressions were applied. Clustering methodology was used to capture correlations of repeated measurements from the same participant to build a median regression model, and bootstrapping (1000×) was applied to correct standard errors of the estimates. The β, linear combination of regression coefficients, represents estimated effect size. Model predictions of % change in outcome variables from semaglutide treatment were calculated from the final fitted models using the formulas 100(e^β^−1) and 100(β/regression intercept) for natural logarithmic (ln)- and nontransformed variables, respectively.

Missing data were assumed to be random; thus, no adjustments were made for missingness in analyses. All analyses were performed using Stata 18.0 and R 4.3.0 statistical software.

## RESULTS

From 10 June 2019 to 28 July 2022, 154 participants were screened and 108 were randomized to semaglutide or placebo (Figure 1). A total of 92 participants completed the 32-week intervention period. During the posttreatment phase, 15 and 13 participants in the semaglutide and placebo groups, respectively, withdrew prior to week 56. As such, 39 and 41 participants, respectively, completed the posttreatment phase. Reasons for discontinuation during the 32-week intervention period have been previously reported [1]. Posttreatment discontinuations were primarily due to loss to follow-up; 1 participant previously assigned to semaglutide died during the follow-up period from a cause unrelated to the study (Figure 1). One participant received semaglutide from a primary care provider for clinical indications, independent of the study protocol, approximately 4 weeks prior to week 56 and was retained in the analysis.

**Figure 1.**
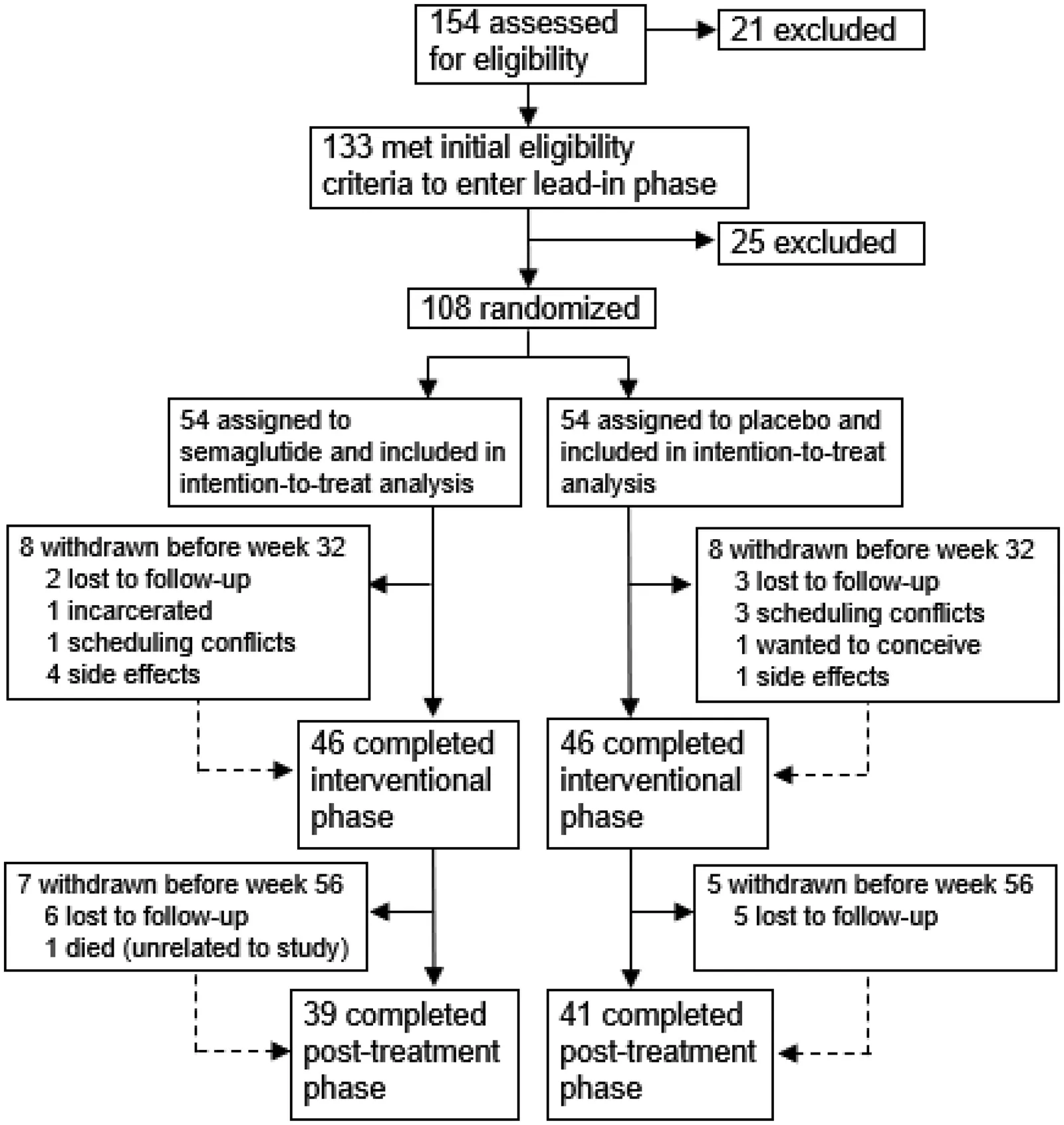
Trial profile.

Baseline characteristics were previously reported and were similar between groups, except for a higher proportion of males in the semaglutide group and higher fasting glucose and triglycerides in the placebo group (Supplementary Table 1). All participants met the prespecified HIV-1 RNA eligibility criterion (<400 copies/mL for ≥6 months) based on screening assessments. At the entry visit following randomization, 1 participant had an HIV-1 RNA of 426 copies/mL, though returned to having a HIV-1 RNA <400 copies/mL on sequent visits without change in regimen. Most participants were receiving integrase strand transfer inhibitor–based regimens, and 74% were on tenofovir alafenamide.

Following discontinuation of semaglutide at week 32, anthropometric measures remained relatively stable in the placebo group. In contrast, the magnitude of semaglutide-associated effects was attenuated at week 56 (Table 1). Body weight increased by 5.8% between week 32 and week 56. As shown in Figure 2, participants were stratified according to the magnitude of weight loss achieved during the intervention period. BMI increased by 5.9% and waist circumference increased by approximately 4.1%, although net reductions compared with baseline were retained in weight, BMI, and waist circumference (Figure 2).

**Figure 2.**
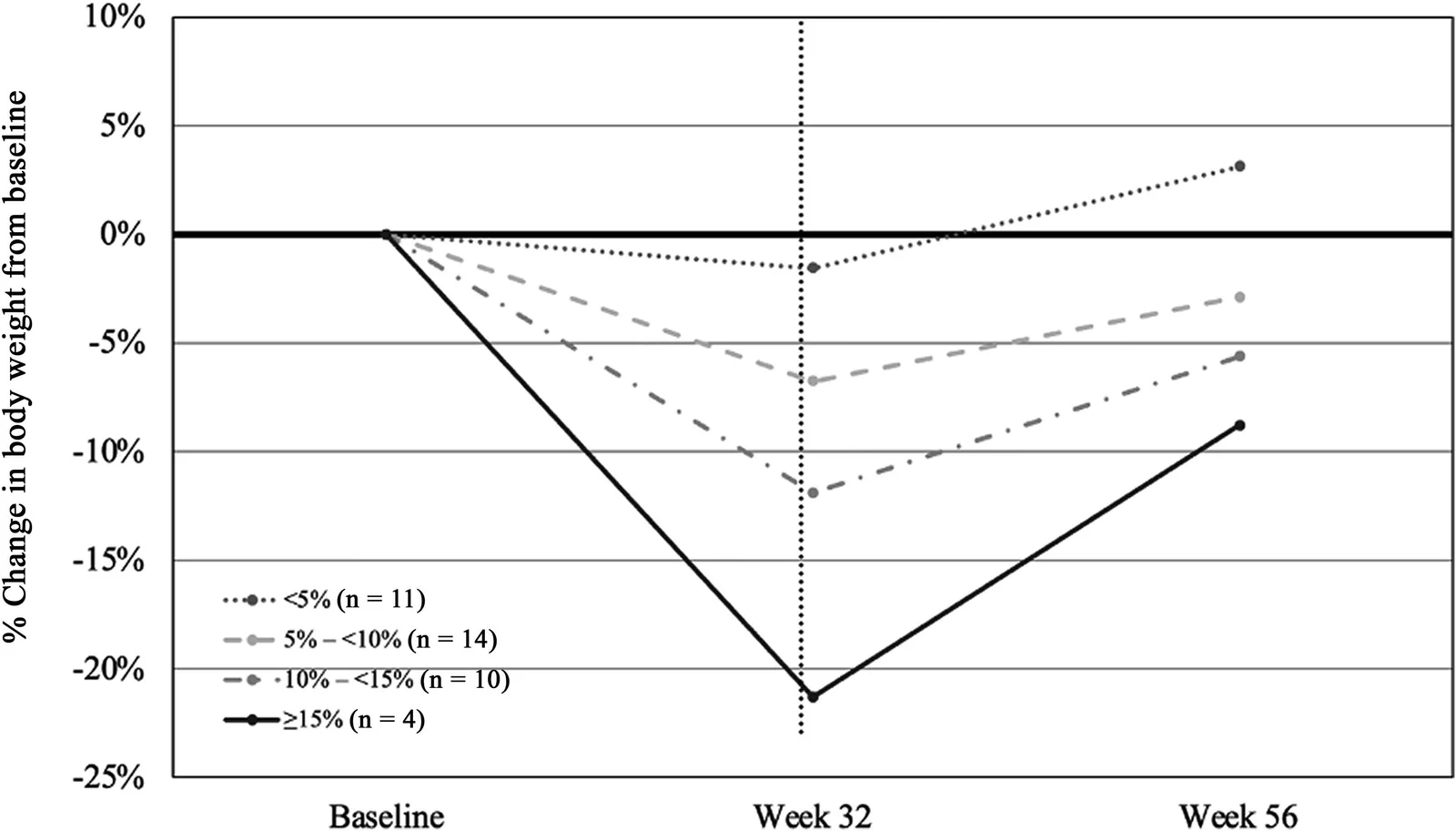
Percentage body weight change from baseline among participants in the semaglutide group based on categorical weight loss during the interventional phase.

**Table 1.** Descriptive Analysis of Demographic and Outcome Variables Over the Study Period^a^

Variable	Baseline		Week 32		Week 56	
	Semaglutide (n = 54)	Placebo (n = 54)	Semaglutide (n = 46)	Placebo (n = 46)	Semaglutide (n = 39)	Placebo (n = 41)
Age, y	48.96 (12.49)	49.52 (11.61)	…	…	…	…
Male sex	38 (70%)	27 (50%)	…	…	…	…
Race or ethnicity						
White	20 (37%)	18 (33%)	…	…	…	…
African American	33 (61%)	34 (63%)	…	…	…	…
Native American	1 (2%)	0 (0%)	…	…	…	…
Biracial	0 (0%)	2 (4%)	…	…	…	…
Hispanic (or Latino)	4 (7%)	5 (9%)	…	…	…	…
Smoking						
Never	25 (46%)	20 (37%)	21 (46%)	19 (41%)	16 (42%)	16 (42%)
Past	14 (26%)	11 (20%)	12 (26%)	8 (17%)	10 (26%)	8 (21%)
Current	15 (28%)	23 (43%)	13 (28%)	19 (41%)	12 (32%)	14 (37%)
CD4 T-cell count, cells/μL	796.26 (416.20)	864.30 (443.22)	804.73 (377.48)	798.47 (376.20)	759.89 (368.28)	811.13 (380.09)
HIV duration, mo	212.99 (115.69)	214.66 (102.99)	220.11 (119.64)	234.72 (94.13)	237.86 (123.77)	246.24 (89.04)
ART duration, mo	170.76 (94.21)	149.63 (78.51)	176.67 (100.58)	168.97 (74.77)	188.20 (89.12)	180.39 (69.17)
Current INSTI use	45 (83%)	43 (80%)	40 (87%)	35 (76%)	32 (82%)	26 (63%)
Current protease inhibitor use	10 (19%)	8 (15%)	8 (17%)	8 (17%)	7 (18%)	6 (15%)
Current tenofovir alafenamide use	43 (80%)	37 (69%)	…	…	28 (93%)	25 (86%)
Weight, kg	100.28 (21.74)	101.17 (20.49)	91.58 (20.47)	100.54 (19.73)	95.05 (20.57)	100.94 (21.90)
BMI, kg/m^2^	33.75 (6.56)	35.61 (7.77)	30.90 (6.35)	35.35 (7.59)	31.93 (5.61)	34.77 (7.15)
Waist circumference, cm	108.62 (12.80)	112.57 (13.36)	103.15 (13.94)	112.33 (13.37)	106.17 (13.55)	111.03 (14.13)
Waist-to-hip ratio	0.97 (0.07)	0.99 (0.06)	0.96 (0.08)	0.97 (0.06)	0.97 (0.08)	0.97 (0.07)
Total body fat, kg	36.07 (12.40)	39.49 (14.02)	31.01 (11.65)	38.24 (13.71)	32.73 (11.76)	37.12 (13.39)
Total trunk fat, kg	19.39 (6.79)	21.33 (7.66)	16.44 (6.46)	20.88 (7.69)	17.85 (6.67)	20.46 (7.71)
Total limb fat, kg	16.68 (6.26)	18.17 (6.87)	14.57 (5.81)	17.37 (6.68)	14.87 (5.79)	16.65 (6.00)
Total lean body mass, kg	59.76 (12.34)	57.69 (11.63)	55.99 (12.10)	57.19 (11.13)	58.07 (12.51)	57.25 (11.71)
Abdominal TAT, cm^2^	414.86 (94.91)	448.31 (101.89)	363.65 (112.08)	444.66 (91.39)	396.52 (110.36)	431.62 (87.18)
Abdominal SAT, cm^2^	299.93 (93.35)	327.14 (106.56)	264.49 (97.15)	319.11 (101.32)	288.53 (102.49)	314.73 (98.89)
Abdominal VAT, cm^2^	114.94 (54.41)	121.18 (47.26)	99.17 (58.32)	125.55 (54.38)	107.98 (60.53)	116.49 (47.48)
VAT:TAT ratio	0.28 (0.12)	0.28 (0.12)	0.27 (0.13)	0.29 (0.13)	0.27 (0.13)	0.28 (0.12)
Pericardial fat, mL	85.89 (45.50)	82.45 (49.88)	85.86 (50.82)	86.10 (49.86)	90.32 (48.78)	87.36 (49.73)
Total right psoas muscle, cm^2^	15.55 (4.91)	14.29 (4.94)	14.50 (4.50)	14.54 (4.87)	15.25 (5.32)	17.25 (10.72)
Liver, HU	56.39 (9.09)	53.58 (10.49)	58.24 (6.10)	53.53 (10.69)	55.48 (9.47)	54.57 (8.97)
Abdominal TAT, HU	−92.08 (4.03)	−91.12 (4.25)	−91.51 (4.72)	−91.86 (5.31)	−92.27 (4.01)	−91.94 (4.29)
Abdominal SAT, HU	−93.89 (4.48)	−92.52 (4.62)	−93.37 (5.05)	−93.20 (5.41)	−93.79 (4.31)	−93.08 (4.56)
Abdominal VAT, HU	−86.24 (5.00)	−86.47 (7.32)	−84.75 (6.61)	−87.68 (6.64)	−86.62 (6.89)	−88.30 (5.45)
Pericardial fat, HU	−108.70 (5.70)	−108.28 (7.28)	−107.83 (7.19)	−109.35 (5.06)	−108.32 (7.66)	−109.90 (1.74)
Total right psoas muscle, HU	46.79 (6.27)	47.06 (6.88)	46.47 (5.75)	47.02 (6.72)	45.56 (6.44)	46.72 (6.07)
HbA1c, %	5.46 (0.55)	5.60 (0.48)	5.08 (0.47)	5.56 (0.44)	5.46 (0.63)	5.54 (0.47)
Fasting glucose, mg/dL	100.61 (10.59)	108.28 (14.87)	102.17 (9.74)	106.60 (14.98)	107.21 (13.36)	109.44 (15.86)
Fasting insulin, μIU/mL	13.76 (11.27)	19.50 (15.41)	16.70 (14.03)	15.89 (9.02)	16.92 (20.63)	18.78 (14.24)
Fasting HOMA-IR	3.47 (2.95)	5.42 (4.81)	4.34 (4.08)	4.23 (2.51)	4.70 (6.06)	5.23 (4.37)
2-h OGTT glucose, mg/dL	114.68 (33.19)	125.98 (56.21)	96.38 (20.46)	117.51 (40.33)	109.72 (32.97)	110.00 (34.06)
2-h OGTT insulin, μIU/mL	51.57 (52.90)	72.61 (74.89)	52.80 (60.49)	71.73 (71.06)	52.34 (85.71)	51.16 (48.45)
2-h OGTT HOMA-IR	16.77 (21.49)	26.16 (33.84)	14.27 (19.47)	23.76 (27.96)	15.52 (24.49)	16.18 (22.32)
Total cholesterol, mg/dL	183.30 (40.96)	185.96 (39.39)	165.09 (35.42)	173.98 (39.83)	175.95 (43.55)	169.02 (42.53)
High-density lipoprotein, mg/dL	48.65 (13.06)	45.03 (11.13)	46.29 (12.11)	43.99 (9.36)	50.74 (12.35)	44.88 (9.79)
Low-density lipoprotein, mg/dL	107.28 (36.47)	108.30 (34.92)	93.87 (32.27)	98.60 (35.13)	99.03 (39.97)	94.56 (37.51)
Very low-density lipoprotein, mg/dL	27.44 (16.09)	29.61 (13.61)	24.96 (13.29)	28.66 (12.97)	26.31 (15.75)	28.75 (12.01)
Triglycerides, mg/dL	137.07 (80.61)	168.30 (107.67)	124.59 (66.26)	153.84 (115.50)	131.15 (78.45)	151.29 (75.28)
Heart rate, beats/min	74.19 (14.36)	76.46 (9.02)	76.17 (14.49)	77.11 (12.71)	75.28 (15.30)	77.56 (11.81)
Systolic blood pressure, mm Hg	127.28 (13.04)	128.33 (16.67)	123.91 (13.80)	129.85 (14.92)	131.28 (16.33)	131.41 (17.70)
Diastolic blood pressure, mm Hg	79.81 (7.80)	79.22 (9.56)	78.98 (6.58)	79.93 (9.62)	79.90 (8.38)	80.41 (11.12)
Estimated weekly physical activity						
Low intensity, min	2697.78 (1672.62)	2608.61 (1753.52)	3195.65 (1773.59)	3140.87 (1863.51)	3347.37 (1614.38)	3191.05 (1785.83)
Moderate intensity, min	2417.78 (1557.54)	2731.22 (1669.40)	2008.04 (1274.60)	2454.13 (1621.43)	2557.89 (1388.73)	2362.11 (1409.50)
High intensity, min	532.04 (824.57)	322.22 (673.98)	411.20 (729.96)	386.41 (817.56)	326.97 (720.30)	435.79 (643.61)
10-y ASCVD risk estimate, %	6.39 (5.23)	8.23 (8.90)	5.74 (5.16)	8.18 (8.44)	6.36 (3.93)	7.41 (6.63)
Total calories, kcal	1905.60 (2173.54)	1808.24 (957.84)	1542.74 (713.17)	1556.13 (740.93)	1888.96 (779.75)	1539.11 (637.71)

Abbreviations: ART, antiretroviral therapy; ASCVD, atherosclerotic cardiovascular disease; BMI, body mass index; HbA1c, glycated hemoglobin; HIV, human immunodeficiency virus; HOMA-IR, homeostatic model assessment of insulin resistance; HU, Hounsfield unit; INSTI, integrase strand transfer inhibitor; OGTT, oral glucose tolerance test; SAT, subcutaneous adipose tissue; TAT, total adipose tissue; VAT, visceral adipose tissue.

^a^Continuous and categorical variables are summarized as mean ± standard deviation and No. (%), respectively. Baseline characteristics remain the same over the study period.

Cessation of semaglutide was associated with significant reaccumulation of adipose tissue across multiple compartments in the semaglutide group (Table 2). At week 56, total body fat increased by 10.4%, with similar increases in total trunk fat (13.0%) and limb fat (9.5%) compared to week 32 values. Abdominal adipose tissue also increased, including a 9.9% increase in total adipose tissue (TAT), 8.6% increase in subcutaneous adipose tissue, and 15.8% increase in VAT. Despite the rebound, participants in the semaglutide group continued to demonstrate net reductions in total body fat, trunk fat, limb fat, and VAT at week 56 compared with baseline. In comparison, participants in the placebo group demonstrated relatively modest changes in total and regional adiposity at week 56 compared with baseline.

**Table 2.** Effects of Semaglutide (β) and Percent Changes (%)^a^ in Important Biomarkers During the Study Period^b^

Biomarker	First 32 Weeks		Follow-up Period (32–56 Weeks)		0–56 Weeks	
	95% CI of β	%	95% CI of β	%	95% CI of β	%
Anthropometric measurements						
ln (Weight, kg)	−.18, −.02	−9.79	.04, .07	5.76	−.13, .03	−4.60
ln (BMI, kg/m^2^)	−.20, −.06	−12.39	.02, .10	5.86	−.15, −.002	−7.25
ln (Waist circumference, cm)	−.14, −.05	−8.90	.02, .06	4.05	−.10, −.00	−5.20
ln (Waist-to-hip ratio)	−.05, .01	−1.98	−.02, .02	−.03	−.05, .01	−2.01
Whole-body DXA measurements						
ln (Total body fat, kg)	−.35, −.10	−20.03	.06, .13	1.44	−.25, .002	−11.68
ln (Total limb fat, kg)	−.36, −.08	−19.91	.04, .14	9.53	−.28, .01	−12.28
ln (Total trunk fat, kg)	−.39, −.13	−22.96	.09, .15	12.97	−.27, −.01	−12.96
Total lean body mass, kg	−6.03, 2.01	−3.67	1.76, 3.45	4.76	−3.43, 4.61	1.08
CT area and volume measurements						
Abdominal TAT, cm^2^	−110.36, −35.37	−15.31	23.11, 71.24	9.91	−63.91, 12.52	−5.40
Abdominal SAT, cm^2^	−76.64, −9.71	−11.58	11.13, 52.86	8.58	−45.11, 22.74	−3.00
Abdominal VAT, cm^2^	−56.79, −7.01	−31.74	−5.41, 37.21	15.82	−42.78, 10.78	−15.92
ln (VAT:TAT ratio)	−.26, .06	−9.34	−.03, .07	2.40	−.23, .08	−7.17
ln (Pericardial fat, mL)	−.36, .08	−13.05	−.02, .17	7.87	−.29, .16	−6.21
ln (Total right psoas muscle, cm^2^)	−.12, .09	−1.75	−.16, .12	−2.41	−.22, .14	−4.12
CT density measurements						
Liver, HU	.91, 6.82	6.74	−5.64, 2.17	−3.02	−1.21, 5.47	3.72
Abdominal TAT, HU	−1.19, 2.33	−.63	−1.83, .28	.85	−2.01, 1.61	.22
Abdominal SAT, HU	−1.92, 1.83	.05	−1.74, .57	.63	−2.56, 1.30	.68
Abdominal VAT, HU	1.17, 6.03	−4.23	−3.50, .57	1.72	−.41, 4.68	−2.51
Pericardial fat, HU	−.19, 1.79	−.73	−2.06, .86	.55	−1.27, 1.67	−.18
Total right psoas muscle, HU	−2.96, 1.99	−1.04	−1.86, 1.48	−.41	−3.21, 1.86	−1.45
Glucose metabolism and insulin resistance						
HbA1c, %	−.78, −.22	−8.93	.24, .76	8.93	−.23, .23	−.00
ln (Fasting glucose, mg/dL)	−.09, .01	−3.67	−.04, .09	2.36	−.07, .04	−1.40
ln (Fasting insulin, μIU/mL)	−.27, .30	1.33	−.51, .07	−20.02	−.50, .08	−18.96
ln (Fasting HOMA-IR)	−.32, .28	−1.81	−.53, .12	−18.55	−.54, .09	−20.02
2-h OGTT glucose, mg/dL	−32.49, −3.51	−15.52	4.06, 39.94	18.97	−7.85, 15.85	3.45
ln (2-h OGTT insulin, μIU/mL)	−.32, .28	−31.70	−.28, .50	11.61	−.70, .15	−23.77
ln (2-h OGTT HOMA-IR)	−1.09, −.13	−45.59	−.17, .79	36.58	−.80, .21	−25.69
Lipid profiles						
Total cholesterol, mg/dL	−22.22, 8.73	−3.46	4.82, 25.99	7.89	−6.97, 24.28	4.44
ln (High-density lipoprotein, mg/dL)	−.01, .17	8.37	−.03, .12	4.77	.03, .22	13.54
Low-density lipoprotein, mg/dL	−18.27, 9.77	−3.72	.14, 18.53	8.16	−8.82, 18.99	4.45
ln (VLDL, mg/dL)	−.39, −.01	−17.88	−.16, .20	2.26	−.37, .02	−16.03
ln (Triglycerides, mg/dL)	−.43, −.04	−20.92	−.17, .21	2.33	−.42, −.004	−19.08
Vital signs						
Heart rate, beats/min	−5.66, 5.66	.00	−10.60, 8.60	−1.28	−10.47, 8.47	−1.28
ln (Systolic blood pressure, mm Hg)	−.10, −.01	−5.40	−.02, .10	3.99	−.07, .03	−1.63
Diastolic blood pressure, mm Hg	−3.71, 3.71	.00	−6.17, 4.17	−1.27	−5.54, 3.54	−1.27
Cardiovascular disease risk						
ln (10-y ASCVD risk estimate, %)	−.74, −.05	−32.53	−.01, .45	24.33	−.53, .18	−16.11

Abbreviations: ASCVD, atherosclerotic cardiovascular disease; BMI, body mass index; CI, confidence interval; CT, computed tomography; DXA, dual-energy X-ray absorptiometry; HbA1c, glycated hemoglobin; HOMA-IR, homeostatic model assessment of insulin resistance; HU, Hounsfield unit; ln, natural log; OGTT, oral glucose tolerance test; SAT, subcutaneous adipose tissue; TAT, total adipose tissue; VAT, visceral adipose tissue; VLDL, very low-density lipoprotein.

^a^Model predictions of percent change in outcome variables from semaglutide treatment were calculated from the final fitted models using the formulas 100(e^β^−1) and 100(β/regression intercept) for natural logarithmic (ln)- and nontransformed variables, respectively.

^b^Statistical significance was determined by whether the 95% CI of β excludes zero.

DXA-derived lean body mass increased modestly following discontinuation (+4.8%) from week 32 to week 56 but was unchanged from baseline. In contrast, modeled estimates demonstrated no significant change in CT-derived psoas muscle area, although these changes were not statistically significant (−1.75% [95% confidence interval, −.16% to .12%]). Psoas muscle radiodensity also remained stable for both the semaglutide group and placebo at baseline, week 32, and week 56. Semaglutide demonstrated some residual effects on ectopic fat. Despite a 7.9% increase in pericardial fat, participants in the semaglutide group demonstrated a net reduction at week 56 compared to adjusted baseline values. Tissue radiodensity effects, though, were attenuated after treatment cessation. At week 56, liver attenuation decreased by approximately 3.0% (ie, increase in liver fat) in the semaglutide group returning almost to baseline. The placebo group exhibited modest increases in pericardial fat at week 56, although liver attenuation (ie, liver fat) remained unchanged (Table 1).

Improvements in glycemic and metabolic parameters (HbA1c, 2-hour OGTT glucose, 2-hour OGTT insulin, 2-hour OGTT HOMA-IR), were not sustained after semaglutide discontinuation. HbA1c increased by 8.9%, with worsening 2-hour glucose (+19.0%) and insulin resistance (+11.6%) at week 56 compared with week 32 (Table 2), while placebo group measures remained relatively stable (Table 1). Similar trends were noted for lipid parameters (total cholesterol, very low-density lipoprotein, and triglycerides, and increases in high-density lipoprotein) with prior improvements mostly attenuated after semaglutide discontinuation. At week 56, favorable effects of semaglutide were no longer present for systolic blood pressure, heart rate, and 10-year ASCVD risk estimate.

Changes in energy intake and physical activity are summarized in Table 3 and Figure 3. During follow-up, median caloric intake increased in the semaglutide group, exceeding baseline values at week 56, whereas caloric intake in the placebo group remained lower compared with baseline values. Low-intensity physical activity increased in both groups from baseline, with a statistically significant increase observed in the semaglutide group. Moderate-intensity physical activity decreased in the placebo group, whereas at week 56, there was a statistically significant increase in the semaglutide group. No significant changes were seen in the high-intensity physical activity group. Supplementary Figure 1 displays box and whisker plots depicting the distributions and changes in physical activity and energy consumption variables at baseline, week 32, and week 56.

**Figure 3.**
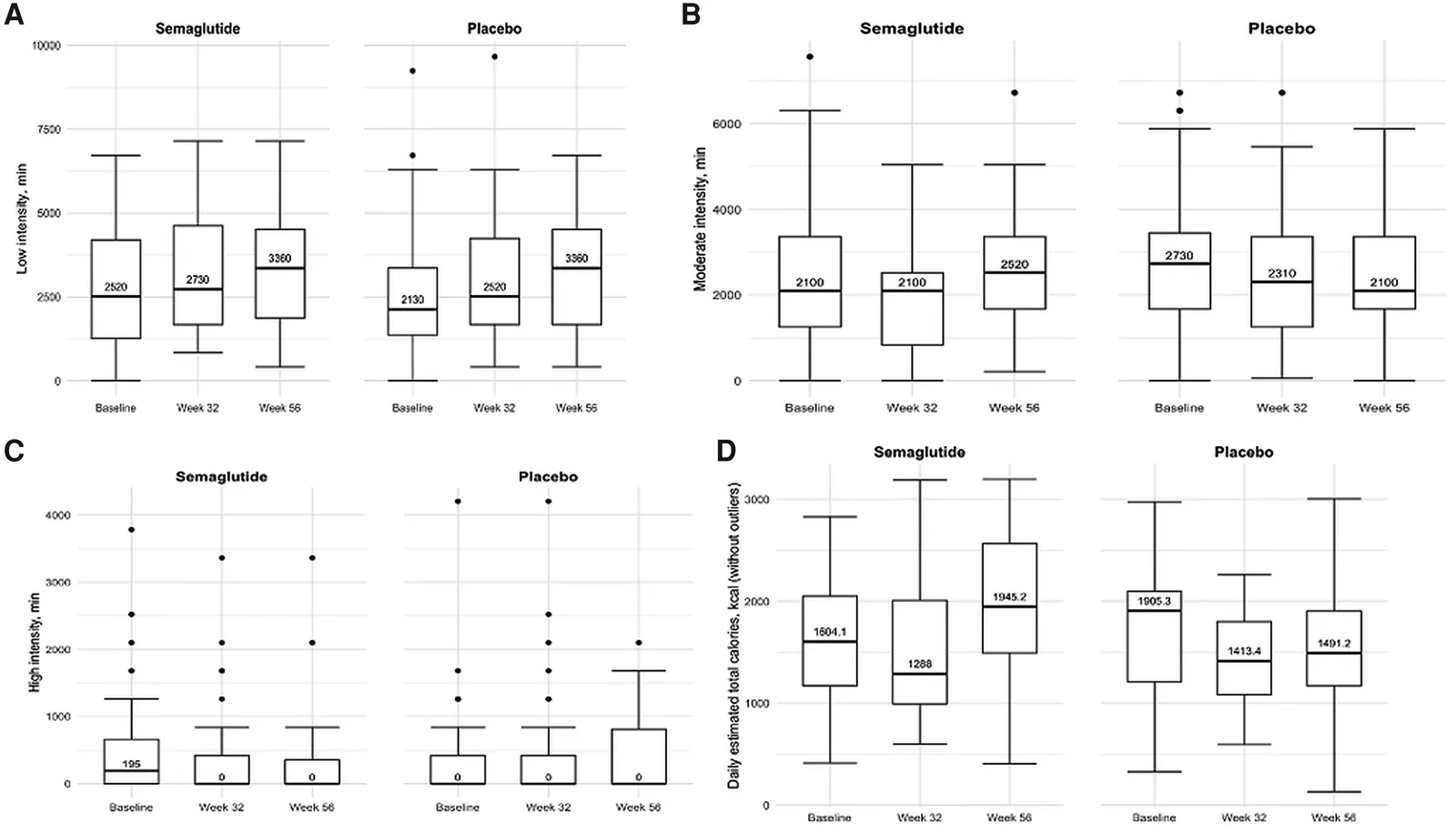
Distributions and changes in physical activity and energy consumption variables over study period. *A*, Low-intensity activity. *B*, Moderate-intensity activity. *C*, High-intensity activity. *D*, Daily caloric intake.

**Table 3.** Relative Changes in Physical Activity and Energy Consumption Over the Study Period

Physical Activity Intensity	Study Group	Week 0 (n = 108)	Week 32 (n = 92)	Week 56 (n = 80)	First 32 Weeks	Follow-up Period (32–56 Weeks)	0–56 Weeks
		Median	Median	Median	M (*P* Value)	M (*P* Value)	M (*P* Value)
Energy, kcal	Placebo	1905.35	1413.45	1491.22	0.94 (.91)	−16.69 (.004)	−6.78 (.14)
	Semaglutide	1604.14	1288.02	1945.20	7.00 (.59)	1.13 (.69)	34.07 (.08)
Low intensity, min	Placebo	2130.00	2520.00	3360.00	0.00 (.13)	0.00 (.47)	20.00 (.09)
	Semaglutide	2520.00	2730.00	3360.00	21.11 (.18)	0.00 (.59)	36.67 (.01)
Moderate intensity, min	Placebo	2730.00	2310.00	2100.00	−25.00 (.23)	0.00 (.75)	−11.11 (.28)
	Semaglutide	2100.00	2100.00	2520.00	−29.17 (.16)	22.22 (.04)	11.43 (.44)
High intensity, min	Placebo	0.00	0.00	0.00	−75.00 (.83)	−33.33 (.77)	−85.71 (.37)
	Semaglutide	195.00	0.00	0.00	−85.71 (.10)	−100.00 (.42)	−100.00 (.43)

Abbreviation: M, relative median percent change.

## DISCUSSION

In our previously reported randomized, placebo-controlled trial, once-weekly semaglutide produced significant reductions in weight, adiposity, and insulin resistance in PWH with HIV-associated lipohypertrophy over 32 weeks. In the present posttreatment phase of the study, discontinuation was associated with partial reversal of these effects over the subsequent 24 weeks. Although modest reductions in weight and adiposity were retained at week 56, substantial reaccumulation of total and regional fat, particularly VAT, was observed. These findings mirror those reported after semaglutide withdrawal in both the HIV and non-HIV populations and underscore the metabolic consequences of GLP-1 discontinuation [2, 3].

Our findings complement the growing literature demonstrating that the beneficial effects of GLP-1RAs on weight and cardiometabolic health are at least partially reversed following treatment discontinuation. In the STEP 1 trial extension, participants regained nearly two-thirds of their prior weight loss during the year following semaglutide withdrawal, accompanied by deterioration in cardiometabolic risk factors [2]. Similarly, Erlandson et al observed weight regain and increases in waist circumference and fasting glucose during 24 weeks following semaglutide discontinuation in PWH [3]. Our findings extend these observations to PWH with central adiposity and provide detailed characterization of changes in regional adipose tissue and body composition following treatment withdrawal.

Although HIV-associated lipohypertrophy has historically been considered distinct from generalized obesity, contemporary PWH may have overlapping contributions from HIV- and ART-associated metabolic alterations, traditional obesity risk factors, and generalized weight gain. The clinical criteria used in this study identified participants with prominent central adiposity arising after ART initiation but cannot definitively distinguish HIV-associated lipohypertrophy from coexisting generalized obesity. Importantly, the pattern observed following semaglutide discontinuation was broadly consistent with studies in populations without HIV, with regain of weight and adiposity and attenuation of metabolic improvements. These findings extend existing GLP-1RA withdrawal data to PWH and suggest that the need for continued pharmacologic exposure to maintain benefit may similarly apply to this population [2, 4, 5].

Beyond overall weight regain, the pattern of adipose tissue reaccumulation observed after semaglutide discontinuation is notable and provides insight into the distribution of tissue reaccumulation after semaglutide withdrawal. In our prior study, semaglutide treatment was associated with marked reductions in total body fat, trunk fat, and visceral adiposity. However, in the current analysis, fat regain after withdrawal occurred across multiple compartments, including total body, trunk, limb, subcutaneous, and visceral depots. Although visceral adipose tissue increased substantially during follow-up, the VAT:TAT ratio remained relatively unchanged, suggesting proportional reaccumulation of abdominal adipose tissue rather than preferential visceral fat regain. This distinction is clinically relevant because changes in body weight alone may not fully characterize the cardiometabolic consequences of GLP-1RA withdrawal. The persistence of some reductions in regional adiposity relative to baseline at week 56 further suggests that reversal was incomplete after 24 weeks, although longer follow-up is needed to determine whether these residual benefits persist.

An important observation was the discordance between DXA- and CT-derived measures of lean tissue following semaglutide discontinuation. Based on DXA, lean body mass increased during the posttreatment period, accounting descriptively for approximately 60% of the observed weight regain. In contrast, modeled analyses demonstrated no significant change in CT-derived psoas muscle area. The reasons for this difference are not entirely clear; however, DXA-derived lean mass should not be interpreted as equivalent to skeletal muscle mass, as it includes nonadipose soft tissues and can be influenced by changes in hydration and organ mass [6, 7]. Therefore, increase in DXA-derived lean mass cannot be assumed to represent recovery of skeletal muscle. Given the descriptive nature of these data and the absence of statistically modeled estimates for compartment-specific regain, this observation should be interpreted with caution. The discordance between these measures highlights the need for further studies incorporating direct assessments of skeletal muscle mass and function to determine the composition and clinical implications of weight regain following GLP-1RA discontinuation.

Glycemic improvements were also attenuated following semaglutide withdrawal, consistent with prior GLP-1RA discontinuation studies. Although some glycemic measures (fasting glucose and fasting HOMA-IR) exceeded baseline values descriptively, this pattern was not consistent across outcomes or uniformly supported by model-based estimates and should therefore be interpreted cautiously. In addition to biological mechanisms, behavioral factors likely contributed to the observed changes following semaglutide discontinuation. Notably, caloric intake appeared to increase during the posttreatment period among participants previously treated with semaglutide, although the change was not statistically significant. Loss of GLP-1–mediated appetite suppression may contribute to weight regain after withdrawal, but behavioral measures were not experimentally controlled, and these findings should be considered exploratory. Taken together, these findings support an emerging chronic treatment model for GLP-1RA therapy, in which continued pharmacologic exposure may be necessary to maintain improvements in weight, adiposity, and metabolic health. Future studies should determine whether specific clinical or body composition characteristics can identify individuals most likely to maintain benefit after GLP-1RA withdrawal and whether adjunctive behavioral or pharmacologic interventions can mitigate weight and adipose tissue regain.

This study has several important strengths. First, the randomized, placebo-controlled design of the parent trial provides a rigorous framework for evaluating posttreatment changes and the effects of semaglutide discontinuation. The longitudinal assessment of glycemic, lipid, and cardiovascular risk markers enables a comprehensive evaluation of metabolic durability after treatment cessation. Last, the follow-up beyond the active treatment phase provides critical knowledge on management of HIV-associated lipohypertrophy. Several limitations should also be acknowledged. Although participants met prespecified clinical criteria for HIV-associated lipohypertrophy, these criteria cannot definitively distinguish HIV- or ART-associated changes in fat distribution from generalized obesity. These phenotypes increasingly overlap in contemporary PWH, and both may have contributed to the adiposity observed in this cohort. Additionally, the reduced sample size of participants who completed the posttreatment phase at week 56 may limit generalizability, although loss to follow-up occurred in both treatment groups and baseline characteristics of those who completed follow-up were similar to the overall cohort. Furthermore, the reduced sample size limits the power to detect between-group differences during the posttreatment phase. The duration of the posttreatment phase was limited to 24 weeks; as such, it is not known if the trajectories of weight regain, adipose reaccumulation, and metabolic outcomes would have stabilized or continued beyond 1 year. Last, behavioral factors such as dietary intake, physical activity intensity, and adherence to lifestyle recommendations were assessed but not controlled experimentally, limiting the ability to determine if observed findings were solely due to medication withdrawal or to other biological and behavioral contributors.

In conclusion, discontinuation of semaglutide in PWH with HIV-associated lipohypertrophy was followed by partial reversal of improvements in weight, regional adiposity, and metabolic health. While a net benefit was observed up to 24 weeks after treatment discontinuation in some anthropometric and body composition parameters, the rebound in adiposity and metabolic parameters raises important questions about the durability of these retained benefits in the absence of ongoing therapy. Taken together, management of HIV-associated lipohypertrophy likely requires a chronic disease model with a multimodal approach of sustained pharmacotherapy and potentially combined with structured behavioral interventions to maintain long-term cardiometabolic benefit.

## Supplementary Material

ofag631_Supplementary_Data

## Supplementary Data

Supplementary materials are available at *Open Forum Infectious Diseases* online. Consisting of data provided by the authors to benefit the reader, the posted materials are not copyedited and are the sole responsibility of the authors, so questions or comments should be addressed to the corresponding author.

## References

[1] Eckard AR, Wu Q, Sattar A, et al Once-weekly semaglutide in people with HIV-associated lipohypertrophy: a randomised, double-blind, placebo-controlled phase 2b single-centre clinical trial. Lancet Diabetes Endocrinol 2024; 12:523–34. 10.1016/S2213-8587(24)00150-5 PMC11417641 38964353

[2] Wilding JPH, Batterham RL, Davies M, et al Weight regain and cardiometabolic effects after withdrawal of semaglutide: the STEP 1 trial extension. Diabetes Obes Metab 2022; 24:1553–64. 10.1111/dom.14725 PMC9542252 35441470

[3] Erlandson KM, Kitch DW, Kantor A, et al The bounce-back effect: what happens after cessation of low-dose semaglutide in people with HIV. Clin Infect Dis 2026; 82:e544–8. 10.1093/cid/ciaf652 PMC13017447 41299212

[4] Quarenghi M, Capelli S, Galligani G, Giana A, Preatoni G, Turri Quarenghi R. Weight regain after liraglutide, semaglutide or tirzepatide interruption: a narrative review of randomized studies. J Clin Med 2025; 14:3791. 10.3390/jcm14113791 PMC12155999 40507553

[5] Rubino D, Abrahamsson N, Davies M, et al Effect of continued weekly subcutaneous semaglutide vs placebo on weight loss maintenance in adults with overweight or obesity: the STEP 4 randomized clinical trial. JAMA 2021; 325:1414–25. 10.1001/jama.2021.3224 PMC7988425 33755728

[6] Abe T, Patterson KM, Stover CD, Young KC. Influence of adipose tissue mass on DXA-derived lean soft tissue mass in middle-aged and older women. Age (Dordr) 2015; 37:9741. 10.1007/s11357-014-9741-1 PMC4315776 25649708

[7] Moncada-Jiménez J, Chacón-Araya Y, Carpio-Rivera E. The effect of passive and exercise-induced dehydration on body composition using dual energy X-ray absorptiometry. Clin Nutr ESPEN 2025; 69:333–41. 10.1016/j.clnesp.2025.07.011 40695407

